# Bipolar Disorder and Cardiovascular Risk in Rural versus Urban Populations in Colombia: A Comparative Clinical and Epidemiological Evaluation

**DOI:** 10.5334/aogh.3479

**Published:** 2021-11-18

**Authors:** Juan Pablo Forero, Alexander Ferrera, Jose Daniel Castaño, Sergio Ardila, Tanya Mesa, Dean Hosgood, Eugenio Ferro

**Affiliations:** 1 Albert Einstein College of Medicine (AECOM), 1300 Morris Park Ave, Bronx, NY, USA 10461; 2 E.S.E. Hospital San Bernardo, Cll. 9 Cra. 5, Filadelfia, Caldas, Colombia; 3 Faculty of Health Sciences, E.S.E. Hospital Universitario de Caldas, Cll. 48 #25-71, Manizales, Colombia; 4 Instituto Colombiano del Sistema Nervioso, Cll. 134 #17-71, Bogota D.C., Colombia

## Abstract

**Background:**

Various multifactorial elements may contribute toward the urban and rural disparities in cardiovascular disease (CVD) risk, particularly among patients with psychiatric diseases.

**Objective:**

To investigate whether rural patients diagnosed and treated for Bipolar Disorder (BD) have different risk profiles and outcomes of CVD compared to urban (BD) patients.

**Methods:**

We conducted a case-control study that included 125 BD patients (cases) from rural Filadelfia, Colombia and 250 BD patients (controls) treated in Bogotá, Colombia. Cases and controls were 2:1, matched by age and sex. We applied the Framingham Heart Study (FHS) risk calculator to assess risk. Differences by rural/urban status (i.e., case-control status) were assessed by chi-square, paired t-tests, and logistic regression.

**Findings:**

Rural BD patients were found to have lower education (p = 1.0 × 10^–4^), alcohol consumption (p = 3.0 × 10^–4^), smoking (p = 0.015), psychiatric (p = 1.0 × 10^–4^) and CV family history (p = 0.0042) compared to urban BD patients. Rural BD patients were 81% more likely to have a more favorable CVD risk profile (OR: 0.19, 95% CI [0.06–0.62]) than urban BD patients, despite rural BD patients having increased CVD morbidity (p = 1.0 × 10^–2^).

**Conclusion:**

Based on increase in morbidity but lower predictive risk in the rural population, our study suggests that the FHS-CVD calculator may not be optimal to assess CVD risk in this population.

## Introduction

Previous research has demonstrated a well-documented discrepancy between urban and rural access to healthcare on a global scale [1]. Many of these studies investigate the differences between the two general populations but fail to highlight the significant stratification within vulnerable subgroups, such as psychiatric patients.

Many studies point to a link between psychiatric diseases and the incidence of cardiovascular disease (CVD) [2,3,4]. This may be due to neuroendocrine or immuno-inflammatory abnormalities underlying bipolar disorder (BD) as well as adverse effects of antipsychotic medications on the cardiovascular system [5,6]. Furthermore, systemic factors imposed by rural versus urban populations are important to consider in this CVD association, including adverse lifestyle factors, limited access to healthcare and education, lack of specialized medical care and absence of continuity of care for rural populations. Additionally, previous research in the general population (non-psychiatric groups) has highlighted that although people with a lower level of education in low-income and middle-income countries have higher incidences of and morbidity from CVD, they have better overall risk factor profiles [7].

In BD, the pharmacological treatments that are used for the disease management in its different clinical phases have been linked to an increased risk of metabolic syndrome [8]. In particular, the second-generation antipsychotics commonly used in BD also contribute to CVD risk. This could explain the presence of CVD as a comorbidity of these patients; although it may not be the only factor involved, as evidenced by literature which links the complex genetic interactions between BD and CVD [9]. One study calls attention to an increased rate of hypertension (HTN) amongst BD patients in Aranzazu, a small rural municipality in Caldas, Colombia (population: 9854) [10], when compared to a matched urban population [11]. Similarly, investigations point to an increased risk of HTN in BD patients compared to other mental disorders, such as schizophrenia [12,13].

Filadelfia (population: 9,630) [10], situated ten miles west of Aranzazu, is a rural municipality also located in the Caldas region of Colombia, famously known for its production of coffee. This is the home of the “Paisa” population, a genetically and culturally homogeneous population of Colombia which has been the focus of genetic studies in neuropsychiatric disorders for the last decade [11]. The rural population, predominantly of Paisa descent, in this district of Colombia is highly affected by major mental disorders, local investigators have discovered high rates of BD type I and II, where its prevalence is estimated to be between 5–8% compared to 1–2% around the world [14].

Filadelfia serves as an appropriate model for investigating whether patients diagnosed and treated for BD in rural populations have different risk profiles and outcomes of CVD compared to patients diagnosed and treated in urban centers. Our group chose to investigate whether there were differences in the risk profiles predictive of CVD and ensuing cardiovascular morbidity in rural versus urban BD patients in Colombia. This study is expected to contribute to the scientific literature, currently limited about CVD risk in rural BD patients. Additionally, it intends to draw attention to national and international authorities responsible for supervising and allocating health resources for primary and secondary care for this group of vulnerable BD patients.

## Materials and Methods

We conducted a case-control study in two different provinces of Colombia to explore the hypothesis that CVD morbidity and psychiatric treatment was different in rural compared to urban BD patients.

Briefly, all BD type I and II patients over age 18 from Filadelfia, Colombia with medical records available were eligible for inclusion. Cases, assumed to be predominantly of Paisa origin, were identified from a review of medical records of subjects diagnosed with BD treated between January 2013 and December 2018 of the San Bernardo Hospital in Filadelfia, Caldas (Colombia). Based on these criteria, 143 total cases from Filadelfia were originally collected and 13 patients were removed from the study due to exclusion criteria or insufficient available medical records (***Figure 1a***). One hundred and twenty-five of 143 cases (87%) were enrolled into the study. Eligible controls (urban) were selected from patients (n = 250) diagnosed with BD treated at Instituto Colombiano del Sistema Nervioso (ICSN) – Clinica Montserrat, a tertiary referral medical center for psychiatry. Controls were 2:1 individually matched to cases based on age (±2 years) and sex (***Figure 1b***). Criteria of exclusion omitted rural cases with residence in urban centers, urban controls with residence in rural zones and any patient with medical history not available prior to 2009. The data was reviewed and collected by five trained investigators from electronic medical records. This research protocol was approved by the Einstein IRB, an independent ethical review committee in Bogota, Colombia, as well as a review committee in Filadelfia, Caldas, Colombia. Requirements for informed consent were waived as the study was a retrospective study based on medical chart review.

**Figure 1a:**
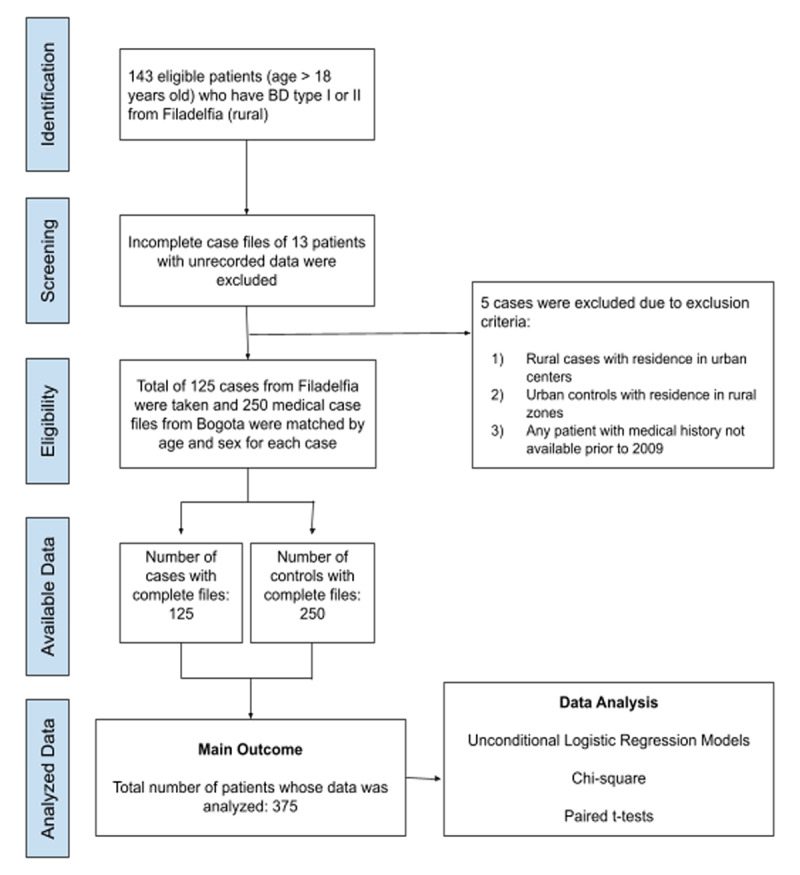
Diagram of case-control research methodology.

**Figure 1b:**
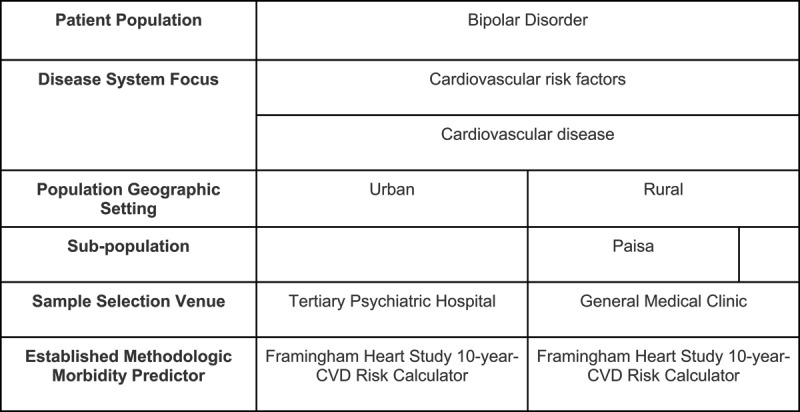
Table delineating differences in geographic setting, subpopulations, and selection venue between urban and rural cohorts studied with Bipolar Disorder.

The study recorded medical histories of cardiovascular diseases, and other existing comorbidities, from medical chart reviews at each institution. Comorbidities were tabulated based on organ system and description. The Framingham Heart Study (FHS) risk calculator was used to assess CVD risk among study populations, which computes 10-year risk of developing CVD based on sex, age, systolic blood pressure (SBP), treatment for HTN, Diabetes Mellitus (DM), smoking, and BMI. The FHS risk score calculator was used to determine either Low (<10%), Moderate (>10%) or High (>20%) risk of CVD. Per recommendations from FHS risk calculator developers, BMI was used instead of cholesterol values to determine FHS calculator risk score due to incomplete lipid profiles upon medical chart review [15].

First, unconditional logistic regression was used to estimate the odds ratio and calculate the 95% confidence interval for the association between CVD risk adjusted for education. Odds ratios and 95% confidence intervals were calculated and adjusted for age and sex. Second, the study repeated the unconditional regression model removing education to determine direction of association and difference in significance. Third, based on variables deemed clinically relevant, the data set was analyzed with multiple univariate and multivariate unconditional regression models analyzing CVD risk: (1) modeling CVD risk profile for the rural BD population when compared with urban BD population, (2) modeling better risk profiles for the urban BD population, (3) modeling better risk profiles for the rural BD population, (4) modeling patients with low risk profiles when compared to patients with a high risk profile in BD patients from Bogota, and (5) modeling patients with low risk profiles when compared to patients with high risk profiles in BD patients from rural Colombia. All statistical methods were performed using JMP and SAS software, version 9.1 (SAS Institute, Cary, NC), unless stated elsewhere.

## Results

Urban BD cases tended to be more likely to complete high school (p = 1.0 × 10^–4^), smoke (p = 3.0 × 10^–4^), and consume alcohol (p = 0.015), than rural BD cases (***Table 1***). Differences were also noted between the populations with an increased likelihood of there being psychiatric (p = 1.0 × 10^–4^) and cardiovascular family history in the urban population (p = 0.0042). We also found that the rural population was more likely to have a GI diagnosis (p = 0.0027), and the urban population was more likely to have a diagnosis of hypothyroidism (p = 1.0 × 10^–4^) (***Table 1***).

**Table 1:** Demographic information comparing Rural Filadelfia Bipolar Disorder population with urban Bogota Bipolar Disorder Population. Comparison of age, sex, BMI, education, alcohol use, current tobacco use, family history, and non-psychiatric comorbid conditions.


DEMOGRAPHIC CHARACTERISTICS	RURAL		URBAN		TOTAL		p-VALUE
		
	n	% OR AVG	n	% OR AVG	n	% OR AVG	

Male	46	36.8	92	36.8	138	36.8	1.0

Age	125	53.2	250	54.1	375	53.8	1.0

BMI	123	25.9	218	26.1	341	26.0	0.73

Completed Education	19	22.1	218	90.5	237	72.5	1.0 × 10^–4^

Alcohol	7	5.98	49	20.9	56	15.9	3.0 × 10^–4^

Smoking	24	20.5	84	33.6	108	29.4	0.015

Psychiatric Family History	27	24.6	156	43.5	183	50.8	1.0 × 10^–4^

CVD Family History	30	28.3	108	44.6	138	39.7	0.0042

Pulmonary Conditions	6	4.80	26	10.4	32	8.53	0.067

GI Conditions	34	27.2	36	9.60	70	18.7	0.0027

Hypothyroidism	8	6.40	55	22.0	63	16.8	1.0 × 10^–4^


Rural/urban differences were also observed in both psychiatric and non-psychiatric pharmacological treatment. The urban population was more likely than the rural population to be treated with Lithium (p = 2.0 × 10^–4^), Lamotrigine (p = 1.0 × 10^–4^), and 1^st^ & 2^nd^ generation antipsychotics (p = 1.0 × 10^–4^), (p = 1.0 × 10^–4^). Conversely, the rural population was more likely to be treated with Valproic Acid (p = 1.0 × 10^–4^) and Selective Serotonin Reuptake Inhibitors (SSRIs) (p = 1.0 × 10^–2^). Our comparison of non-psychiatric medical management finds that the only statistical difference is in NSAID use, which is more likely to be used in the urban population (p = 0.012) (***Table 2***).

**Table 2:** Differences in psychiatric and non-psychiatric pharmacological treatment between rural (Filadelfia) and urban (Bogota) Bipolar Disorder populations in Colombia.


PHARMACOLOGICAL TREATMENT	RURAL		URBAN		TOTAL		p-VALUE
		
	n	%	n	%	n	%	

Valproic Acid	84	67.2	115	46.0	199	53.1	1.0 × 10^–4^

Lithium	17	13.6	79	31.6	96	25.6	2.0 × 10^–4^

Lamotrigine	1	0.8	37	14.8	38	10.1	1.0 × 10^–4^

Anticonvulsants	13	10.4	22	8.80	35	9.33	0.62

1st Generation Anti-Psychotics	17	13.6	116	46.4	133	35.5	1.0 × 10^–4^

SSRIs	52	41.6	71	28.4	123	32.8	1.0 × 10^–2^

Atypical Antidepressants	25	20.0	34	13.6	59	15.7	0.11

2nd Generation Anti-Psychotics	47	37.6	224	89.6	271	72.3	1.0 × 10^–4^

Lipid Drugs	24	19.2	42	16.8	66	17.6	0.57

Diabetes	9	7.20	19	7.60	29	7.47	0.89

Anti-Coagulants/Platelets	8	6.40	8	3.20	16	4.27	0.15

NSAIDs	40	32.0	114	45.6	154	41.1	1.0 × 10^–2^


The burden of cardiovascular disease was higher in the rural population, particularly in peripheral artery disease (PAD) (p = 1.0 × 10^–4^) (***Table 3***), compared to the urban population. The urban patients had a higher overall SBP (p = 3.0 × 10^–4^) and were more likely to smoke than the rural patients (p = 0.015). On the other hand, the rural population was more likely to have a diagnosis of HTN (p = 0.0064) and dyslipidemia (p = 1.0 × 10^–4^). A higher proportion of the urban population was high risk, but this was not statistically significant (p = 0.16) (***Table 3***).

**Table 3:** Comparison of overall Cardiovascular Disease morbidity, risk factors and profiles between rural (Filadelfia) and urban (Bogota) patients with Bipolar Disorder in Colombia.


CVD MORBIDITY	RURAL		URBAN		TOTAL		p-VALUE
		
	n	%	n	%	n	%	

Overall	21	16.8	20	8.00	41	10.9	1.0 × 10^–2^

CAD	10	8.00	9	3.60	19	5.07	0.067

PAD	15	12	3	1.20	18	4.80	1.0 × 10^–4^

CHF	0	0	4	1.60	4	1.07	0.16

Arrhythmia	2	1.60	3	1.20	5	1.33	0.75

Valvular	0	0	1	0.40	1	0.26	0.48

Stroke/TIA	1	0.80	2	0.80	3	0.80	1.0

CVD Risk Factors for FHS							

SBP	119	118	244	123	363	121	3.0 × 10^–4^

Treatment for HTN	39	31.2	58	23.2	97	25.9	0.095

HTN	38	30.4	45	18.0	83	22.1	0.0064

Diabetes	12	9.60	18	7.20	30	8.00	0.42

Smoking	24	20.5	84	33.6	108	29.4	0.015

BMI >30	20	16.7	45	20.6	65	19.2	0.37

Dyslipidemia	55	44.0	30	12.0	85	22.7	1.0 × 10^–4^

Estimated CV Risk Prediction	95	9.72	206	11.3	301	10.8	0.15

>20% 10 yr Risk of CVD	13	13.7	42	20.4	55	18.3	0.16


Rural patients had a better CVD risk profile compared to urban controls (OR: 0.19 95% CI: 0.06–0.62) (***Table 4***). The rural population is less likely to have completed education, (OR: 0.012 95% CI: 0.003–0.045), and less likely to be treated with 1^st^ generation (OR: 0.12 95% CI: 0.029–0.462) and 2^nd^ generation (OR: 0.05 95% CI: 0.01–0.16) antipsychotics, respectively.

**Table 4:** Cardiovascular Disease (CVD) risk profile (>10% risk of CVD in 10 years), education, alcohol consumption, and psychiatric treatment for the rural (Filadelfia) Bipolar Disorder (BD) population when compared with an urban (Bogota) Bipolar Disorder population in Colombia.


	ODDS RATIO	95% CONFIDENCE INTERVAL

CVD Risk	0.192	0.059–0.623

Completed Education	0.0120	0.003–0.045

Alcohol Consumption	0.191	0.032–1.135

1st Generation Anti-psychotics	0.115	0.029–0.462

2nd Generation Anti-psychotics	0.0460	0.013–0.156

Valproic Acid	1.957	0.611–6.274

Lithium	1.74	0.472–6.435

Atypical Antidepressants	1.21	0.272–5.382

SSRI	1.53	0.485–4.847


When modeling for factors associated with better risk profiles in both populations, our results suggested that those with better risk profiles in Bogota were less likely to be treated with Valproic Acid (OR: 1.957 95% CI: 0.611–6.274), and more likely to be treated with Atypical Antidepressants (OR: 1.21 95% CI: 0.272–5.382), though not statistically significant (***Table 5***). Additionally, those with a better risk profile less likely to be receiving lipid-lowering agents (OR: 0.275 95% CI: 0.103–0.734). In Filadelfia, no such relationship existed; however, those with better profiles in the rural population were significantly less likely to have been treated with NSAIDs (OR: 0.248 95% CI: 0.077–0.798).

**Table 5:** Risk profiles associated with <10% risk of Cardiovascular Disease (CVD) in 10 years compared to those with >10% risk of CVD in ten years, among Bipolar Disorder patients in Colombia. Lamotrigine was excluded in the rural population due to small sample size.


	BOGOTA (URBAN)		FILADELFIA (RURAL)	
	
	ODDS RATIO	95% CONFIDENCE INTERVAL	ODDS RATIO	95% CONFIDENCE INTERVAL

Alcohol	0.803	0.359–1.793	0.692	0.054–8.825

1st Generation Anti-psychotics	1.43	0.729–2.794	0.638	0.121–3.369

2nd Generation Anti-psychotics	1.64	0.542–4.957	1.07	0.371–3.081

Lithium	1.22	0.58–2.583	1.35	0.3086.315

Valproic Acid	0.521	0.264–1.03	0.820	0.244–2.763

TCA	1.76	0.132–23.518	1.57	0.191–12.901

SSRI	1.05	0.493–2.228	2.19	0.711–6.728

Atypical Antidepressants	3.09	0.967–9.886	0.605	0.154–2.38

Lamotrigine	1.81	0.699–4.7		

Anticonvulsants	1.08	0.358–3.236	1.39	0.226–8.563

Lipids Medications	0.275	0.103–0.734	1.19	0.248–5.69

Anticoagulants	0.295	0.038–2.303	2.42	0.196–29.871

NSAIDs	0.707	0.63–1.39	0.248	0.077–0.798


In Bogota, patients with an elevated FHS risk score (>20% CVD risk in 10 years) were more likely to be treated with medications for glycemic control (OR: 0.037 95% CI: 0.008–0.167) compared to with patients with risk <10%. Further, high risk profiles were associated with lipid-lowering agents (OR: 0.409 95% CI: 0.153–1.093), though not statistically significant. Conversely, no such differences were found between high risk and low risk patients in rural Filadelfia (***Supplementary Table 1***).

## Discussion

This is the first study, to the best of our knowledge, that compares CVD risk profiles between urban and rural patients with comorbid BD in a middle-income country. Accordingly, as suggested by this analysis, the urban population is more likely to have toxic habits and higher education as well as more likely to be using 1^st^ and 2^nd^ generation antipsychotic medications. Additionally, the rural population showed evidence of better CVD risk profiles as indicated by the FHS risk calculator; however, the same population had a higher morbidity of CVD disease. The implication is that the CVD risk calculators are not accurately predicting risk of CVD in this rural population. This phenomenon of either underestimation or, in the case of US Hispanics, overestimation, has also been noted with this risk calculator; nonetheless it has been validated for global studies in the past [16,17,18].

Demographically speaking, our populations are representative of the general trends between urban and rural subgroups in that the urban population tends to be more educated and has increased access to specialized medical care. Furthermore, we find that the urban population was more likely to use tobacco and drink alcohol, two well known risk factors for CVD [15]. Interestingly, we were surprised to find a higher prevalence of self-reported psychiatric family history in the urban group, given the high prevalence rates of BD in the rural population, this finding could be explained by the social stigma associated with BD.

In terms of comorbid conditions, the authors also noted an increased prevalence of GI disorders in the rural population. Given the fact that this population’s predominant treatment for BD is Valproic acid, and its side effects are closely tied to GI discomfort [19], this could provide a plausible explanation; however, no clear association was elucidated. Next, we find that the urban population is 3.5 times more likely to have a diagnosis for hypothyroidism as compared to the rural population. Given that Lithium is used at almost 2.3 times the rate in the urban population compared to the rural population, this may suggest a role of medication side effect in the population [20].

Beyond the differences in demographics, family history, and comorbidities, some of the starkest differences seen in our populations of interest was in their psychiatric treatment. As mentioned in the introduction, the mainstay treatment for BD in rural communities in Colombia is Valproic Acid or Lithium. The two of these account for most of the psychiatric treatment for these patients (***Table 2***). It was also startling to see that a substantial proportion of the rural patients were being treated with antidepressants (***Table 2***), a well-known yet controversial contraindication to treatment with BD patients [21]. This is likely due to prescriber inexperience, insurance limitations, and lack of access to more novel treatments. Conversely to this population, we see the urban cohort is more likely to be treated with 1st & 2nd generation antipsychotics and Lamotrigine. The prevalent use of antidepressants is also significantly lower than in the rural counterparts (***Table 2***). Interestingly, we presumed that the induction of metabolic syndrome with use of antipsychotics would play a role into CVD of urban patients [22,23], but no such association was noted in our study (***Table 5***). Outside of psychiatric treatment, we only found increased use of NSAIDs in urban patients (***Table 2***). Given recent literature exploring the role of inflammatory markers [24,25] in BD patients, this is a relationship the authors found intriguing.

Literature has suggested a relationship between mental disorders and the onset of hypertension [26]. Focusing in on previous studies in Colombia, these have pointed to social disparities as a driver of hypertension [27]. Furthermore, literature suggests that socioeconomic status can serve as a predictor for CVD in countries like Colombia [28]. Therefore, when analyzing the morbidity of CVD between the two populations, the authors hypothesized an increased burden on the rural population. Based on our analysis, this hypothesis turned out to be true. Particularly specific, PAD was the most comorbid cardiovascular condition in the rural group (***Table 3***). Given a higher occurrence of CVD in the rural population, authors expected to see elevated risk profiles for this population. Surprisingly, when calculating the FHS-CVD risk score for both populations, there was no significant differences found. However, our analysis suggested that the urban population had a higher proportion of high-risk patients than the rural population (***Table 3***). These findings are consistent with published literature on the subject [7].

**Figure 2:**
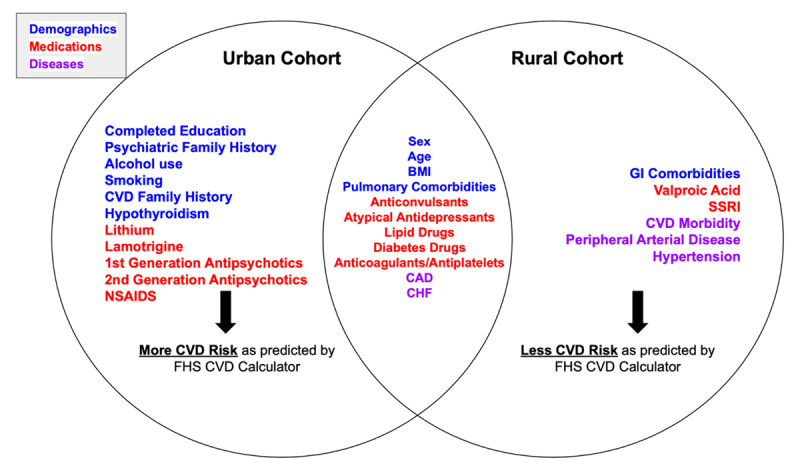
Graphic comparing statistically significant differences and similarities in demographic, pharmacotherapeutic, cardiac comorbid variables, as well as predicted Cardiovascular disease (CVD) risk, between the urban and rural bipolar disorder (BD) cohorts.

Our results suggest the lower CVD risk profiles in the rural population can be explained by the lower prevalence of smoking, and decreased SBP, used by the FHS-CVD risk calculator (***Table 3***). Given the increased CVD morbidity in the rural population, this suggests that these variables used to calculate CVD risk are inadequate in this population sample (***Figure 2***). This finding is consistent with previous rural versus urban studies done in neighboring Venezuela [29]. This coupled with the increased burden of disease in the rural population further supports the inadequacy of risk calculators in rural populations in countries like Colombia. This is consistent with previous studies, comparing non-psychiatric urban and rural cohorts, done in countries of similar income profiles which highlight how contributing modifiable risk factors for CVD vary by a country’s economic level. These studies support the idea that CVD risk calculators lack generalizability when used in rural populations in middle-low-income countries [30,31,32].

Importantly, our results also suggest that factors not accounted for by the risk calculator are accounting for the excess morbidity in the rural population, whose burden of CVD is not predicted by the risk calculator. Previous studies have suggested that the heterogeneity of CVD comorbidity in bipolar disorder patients may be related to variation in the genetic susceptibility to CVD across subgroups of BD patients [12]. Given the potentially unique genetic background of our rural cohort, genetic factors of the Paisa genome may contribute to our observed discrepancies. Other contributing factors may be related to lifestyle habits, socioeconomic background, and comorbid illness. This is in line with previous research on variables not accounted for by CVD risk calculators when predicting risk [33].

Next, patients from the urban population with the best CVD risk profiles (<10% CVD risk) were less likely to be treated with lipid drugs, a finding consistent with current medical literature suggesting the role of dyslipidemias in CVD [15]. Though not significant, but still suggestive, Valproic Acid was used half as much in patients with the best risk profiles in Bogota (***Table 5***). This result provides one plausible explanation for the higher CVD morbidity in Filadelfia that may be linked to Valproic Acid which was more commonly used in the rural population (***Table 2***). Using the same model, in the rural population, we found that the patients in Filadelfia in the low-risk category were less likely to be treated with NSAIDs than their moderate/high risk counterparts (***Table 5***). Again, intriguing given the currently elucidating relationship between BD and inflammation [5,6].

## Limitations

The Paisa population may be a genetic isolate with a potentially unique genetic profile, therefore our generalizability may be limited and not extend to all rural populations. Additionally, it is important to note that mental illness, particularly in rural areas, is extraordinarily stigmatized. If a person seeks out a mental health professional, it is generally viewed with a negative connotation by the rest of the population. This often leads to labels such as “crazies,” or “the crazy town.” This is a factor that prevents people with mental illness from seeking specialized medical care. Given the lack of education in rural towns, patients affected by mental disorders oftentimes attribute their symptoms to part of their personality, hexing or demonic possession. These individuals rarely seek medical care. Another potential limitation was a selection bias toward patients that favor seeking medical attention. In the rural setting, there is only one clinic serving all medical problems in the municipality. Some patients in the municipality were still five to seven hours away walking distance. Because of the extensive effort required to seek medical care in this setting, it can be implied that only those that were highly motivated would seek treatment. The urban cohort may also introduce selection bias since access to care is more widely available, and the patient population is presumably more psychiatrically ill in a tertiary center.

Furthermore, in tabulation of comorbid conditions, we were unable to integrate Cumulative Illness Rating Scales (CIRS), to assess our cohorts due to lack of data availability, future research should better integrate this tool for greater accuracy in measuring these variables. Lastly the power of our study was limited by the number of rural patients included in our study.

## Conclusion

Even though the urban population in Bogota had more favorable risk profiles than the rural Filadelfia population, the rural population had significantly elevated morbidity of CVD. This suggests that the calculator used to gauge risk is a generally inadequate tool to assess CVD risk in rural BD patients in countries like Colombia. Additionally, differences in mainstay psychiatric treatment between rural and urban patients were stark, likely a reflection of the multifactorial variables affecting rural, generally uneducated patients. “The data collected from the study suggests a better method of risk stratification is needed for rural BD populations that is generalizable to other psychiatric populations. Once implemented, treatment needs to then be optimized to address risk factors not captured by the FHS calculator, for both psychiatric and non-psychiatric patients.”

## Supplementary Table

**Supplementary Table 1:** Comparison of unconditional regression models from Bipolar Disorder patients from rural Colombia (Filadelfia) and urban Colombia (Bogota). Modeling is based on patients with low risk profiles (<10% Cardiovascular disease risk in 10 years) when compared to patients with high risk profiles (>10% Cardiovascular disease risk in 10 years). Lamotrigine, TCA, Anticoagulant, and Diabetic Medications were excluded since these variables did not have sufficient variation in the data.


MODELING PROBABILITY FOR PATIENTS WITH BETTER RISK PROFILES	BOGOTA (URBAN)		FILADELFIA (RURAL)	
	
	ODDS RATIO	95% CONFIDENCE INTERVAL	ODDS RATIO	95% CONFIDENCE INTERVAL

Alcohol Consumption	0.742	0.274–2.007	0.44	0.031–6.218

1st Generation Anti-psychotics	1.14	0.487–2.66	0.715	0.117–4.361

2nd Generation Anti-psychotics	0.558	0.118–2.635	1.14	0.299–4.352

Lithium	0.857	0.333–2.201	3.14	0.299–4.352

Valproic Acid	0.721	0.307–1.691	1.08	0.246–4.746

TCA	0.753	0.038–15.047		

SSRI	0.902	0.352–2.308	1.20	0.306–4.678

Atypical Antidepressants	4.47	0.76726.048	1.78	0.308–10.288

Lamotrigine	1.17	0.366–3.713		

Anticonvulsants	1.45	0.33–6.397	0.689	0.107–4.443

Lipid Meds	0.409	0.153–1.093	0.597	0.114–3.131

Diabetic Meds	0.0370	0.008–0.167		

Anticoagulant	0.422	0.052–3.429		

NSAIDs	0.779	0.333–1.822	0.471	0.122–1.819


## Data Accessibility Statement

Data are available upon reasonable request.
